# Informing Parents about Newborn Screening: A European Comparison Study

**DOI:** 10.3390/ijns7010013

**Published:** 2021-02-26

**Authors:** Amber IJzebrink, Tessa van Dijk, Věra Franková, Gerard Loeber, Viktor Kožich, Lidewij Henneman, Marleen Jansen

**Affiliations:** 1 Department of Clinical Genetics, Section Community Genetics, Amsterdam University Medical Center, Vrije Universiteit Amsterdam, 1007 MB Amsterdam, The Netherlands; a.ijzebrink@amsterdamumc.nl (A.IJ.); t.vandijk@amsterdamumc.nl (T.v.D.); l.henneman@amsterdamumc.nl (L.H.); 2 Department of Pediatrics and Inherited Metabolic Disorders, Charles University First Faculty of Medicine and General University Hospital Praque, 128 08 Prague 2, Czech Republic; vera.frankova@lf1.cuni.cz (V.F.); viktor.kozich@vfn.cz (V.K.); 3 Institute for Medical Humanities, First Faculty of Medicine, Charles University Praque, 128 08 Praque 2, Czech Republic; 4 International Society for Neonatal Screening, 3721CK Bilthoven, The Netherlands; gerard.loeber@gmail.com; 5 Centre for Health Protection, National Institue for Public Health and the Environment (RIVM), 3720 BA Bilthoven, The Netherlands

**Keywords:** newborn screening (NBS), informed decision, knowledge aspects, parental information, European information products

## Abstract

Knowledge about newborn screening (NBS) is an important factor for parents to make an informed decision about participation. In Europe, countries inform parents differently about their NBS program, potentially including different knowledge aspects in their information. The aim of this study was to assess twenty-six European parental information products and to analyze their knowledge aspects through a content analysis. The analyzed aspects were compared to a list of eight knowledge aspects from scientific literature. The list includes aspects important for parents’ decision-making, such as the purpose of screening. The study showed that most of the eight knowledge aspects are included in NBS information products of the majority of countries. However, there were differences between countries, for example in the amount of detail and phrasing of the information. Additional relevant knowledge aspects have also been identified and are recommended to optimize information products, such as the handling of residual bloodspot samples. This study only assessed knowledge aspects in information products meant for printing, but many countries also use other communication methods, and the impact on knowledge of the delivery of the information needs further study. Preferences of parents on alternative communication methods need to be considered and evaluated on their effectiveness.

## 1. Introduction

In newborn screening (NBS) programs worldwide, babies are screened for rare, congenital disorders through analysis of a blood sample [1]. NBS has the potential to prevent severe morbidity or even mortality, when properly timed and performed [2,3]. NBS programs have developed from programs that screen for a small number of conditions to complex programs sometimes including over 50 conditions. Countries differ in the programs they have in place. Different interpretations of screening criteria, local politics, healthcare structures, and varying perspectives on how to implement NBS contribute to these program differences [3,4,5,6,7]. As a result, countries differ in the conditions screened, screening methodology, storage of residual specimens, and information provided to parents [6,8]. Here, we will focus on parental information about NBS.

Parental information about NBS plays an important role in public support for NBS [3,8,9,10]. It provides knowledge and transparency about the programs. Despite the availability of parental information, a common challenge in NBS is the lack of knowledge of parents about NBS [9,11,12]. Parents and healthcare professionals often view NBS as a routine procedure, while informed participation is of great importance, and also strived for with informed consent procedures in place in around half of European countries [8,13,14]. For example, parents have the right to know the advantages and disadvantages of screening [15,16,17,18,19]. Moreover, several studies have established a link between poor parental knowledge and distress caused by false-positive test results. This illustrates the importance of information provision and retainment about NBS [20,21], especially since the effects of a false positive result can influence healthcare use throughout time [22].

To promote public support for NBS, information needs to ensure that parents make an informed decision to participate in the program [8,9,19,23]. To give guidance in effective and efficient protocols for clinical and laboratory standards, international NBS consensus and guidelines are encouraged [3]. However, international consensus and guidelines may also be of great importance in informing parents about NBS [8]. Although there are many international differences in NBS programs, there are common elements. These common elements might prove helpful in developing or updating parental information. To define the knowledge needed in information about screening, the General Medical Council (GMC) in the United Kingdom has set up professional guidelines with eight knowledge aspects that are deemed important for informed consent for screening (Table 1). GMC does not give a specific moment when this information should be provided, as long as it is before the screening is performed. We will use their framework in our analyses [17,24]. With this study, we aim to contribute to insights about common elements in parental information about NBS in Europe and how this information is presented by analyzing European information products for parents, by using the GMC’s knowledge aspects.

## 2. Materials and Methods

### 2.1. Data Collection

Parental information products from different European countries were collected by contacting 44 contact persons of NBS programs by e-mail (8 April 2018) through the International Society of Neonatal Screening (ISNS) network. Most countries provide information on a national level; if an information product was regional, we have indicated that in Table 2. While many countries have their information products available in different languages, for analysis purposes, we collected information products in English if an English version was available next to the native version for parents. If the information product was not available in English, it was translated to English using Google Translate. Native speakers found through the personal network of the research team were asked to check the translated information product against the original. Except for a few minor adjustments, all translations were correct according to the native speakers. 

### 2.2. Data Analysis

All information products were analyzed on the type of information product, the language of the delivered information product, and the number of pages. Three types of products were distinguished: an information sheet, a leaflet, and a booklet. An information product was considered an information sheet when the information about NBS was presented to fit on one A4, single or double-sided. A leaflet was defined as a product that folds into three, resulting in six panels for information. Information products were regarded as booklets when they consisted of multiple pages with information.

Open and axial coding were applied using ATLAS.ti software version 8.0. First, open coding was used to assess which main topics were included in the products. Content of the products were described and classified into themes in the codebook, by labeling key words or sentences to avoid misinterpretation between codes. Thereafter, axial coding was applied to categorize all codes within the eight knowledge aspects (Table 1). Per country, it was examined whether they have adequately covered all aspects in their products. Adequately covered was defined as inclusion of half or more of the codes for the relevant knowledge aspect. One exception was made for the fourth knowledge aspect: the uncertainties and risks attached to the screening process (Table 1). If only the reliability of NBS was addressed, the presented information was assessed as adequate on aspect 4. Open and axial coding were both used to be able to identify possible other relevant knowledge items in the information products that are not included in the GMC’s knowledge aspects. 

To reduce interpretation bias of this study, the data were coded by A.I., M.J., and T.D. A.I. coded all the information products, M.J. and T.D. coded four randomly selected products to increase the internal validity of the study. After comparing the codes between the three researchers, the codes were adjusted to contain more detail. 

Ethical approval by the Medical Ethics Committee was not required as public data were used. 

## 3. Results

### 3.1. Response Rate and Sample Characteristics

A total of 29 out of 44 countries responded to the e-mail (response rate 66%). Except for Israel, Kazakhstan, and the Russian Federation, all countries are included in this study (*n* = 26). Israel and Kazakhstan do have online information, but no information product that is distributed in print to parents was obtained. The Russian Federation did send information products, but we were unable to translate them from Russian to English.

Table 2 shows the characteristics of all twenty-six information products collected through the ISNS network. We received one information product per country. Thirteen information products were provided in English, the other thirteen were translated from native language to English. The booklet of the United Kingdom includes not only information about NBS but also general information on screening tests and other prenatal screening tests. In addition to NBS, the Netherlands also has information about the hearing test in the same information product, since these tests are usually done simultaneously. Only relevant pages of both products from the UK and the Netherlands were analyzed and counted. 

### 3.2. Knowledge Aspects in Parental Information Products

The first knowledge aspect: (1) the purpose of NBS, is discussed in all twenty-six information products (Table 3). In addition, the following aspects are described in about 75% of the information products: (2) the likelihood of positive and negative findings, (4) the uncertainties and risks attached to the screening process, (5) medical implications of screening, and (8) follow-up plans including the availability of counselling and support services. The social and financial implications of screening (aspect 6 and 7) were described by twelve and ten countries respectively, and the possibility of false positive and false negative findings (aspect 3) was identified in eight information products.

1. The purpose of screening

All twenty-six countries address the purpose or importance of NBS, usually describing an early diagnosis and treatment of disease. The importance of participation was often addressed in the same section as the purpose of NBS. While they all discussed the purpose of screening, the information products differ in the amount of detail they used.

*“The purpose of the test is to find children with very rare and severe but treatable metabolic diseases. An early diagnosis is crucial to enable good prognosis for the child.”*

(Sweden, information sheet)

*“The drop saves a life.”*

(Romania, information sheet)

2. The likelihood of positive and negative findings

Nineteen countries described the probability of positive and negative results of NBS. The emphasis is often on the chance of a positive result, when abnormal values are found by analysis of the blood sample that may indicate an increased risk of disease. Most products discuss how parents are informed about the results more often than the possible results as such. The information products often describe that parents are informed as soon as possible in the case of a positive result. A negative result is hardly ever communicated to parents.

How countries formulate the possible results varies; some countries write about positive and negative results and others use normal and abnormal results.

*“A small number of babies will screen positive for one of the conditions. This does not mean they have the condition, but they are more likely to have it. They will be referred to a specialist for further tests.”*

(United Kingdom, booklet)

*“If the results are normal, they will not be returned directly to you but will be available in the maternity ward or in the paediatric ward where the sample was taken. If one of the tests shows an abnormal result, you will be quickly informed. A check done as soon as possible will tell if your child really needs to be treated.”*

(France, booklet)

3. The possibility of false positive and false negative findings

A few countries address the chance of false positive or false negative findings in NBS. Some countries explicitly discuss the term of false positive and false negative, whereas other countries mention that there is still a chance that the baby is healthy when there is a positive result of NBS, however, additional tests are necessary to diagnose a disease.

*“There is a slight probability that the laboratory tests on the blood taken from the heel prick indicate an abnormality while further tests in hospitals show that your child does not have the disease in question. This is regrettable but unavoidable.”*

(Netherlands, booklet)

4. The uncertainties and risks attached to the screening process

This knowledge aspect is strongly related to the possibility of a false positive or false negative result (aspect 3). However, that aspect describes only the probability of a false positive or false negative result of the conditions that are screened for. The uncertainties and risks outline the reliability of the test in general and the possible need for a repeated heel prick.

Twenty countries mention the possible uncertainties and risks associated with the NBS program. The Netherlands describe that NBS cannot guarantee that there is nothing wrong with a child; other countries, like the United Kingdom, indicate that the test is not perfect. Additionally, some countries mention reasons for a repeated test, for example, insufficient quality of the blood sample or an unclear test result.

*“Unclear result: A blood sample needs to be collected again, usually because the first sample was not collected properly, or was taken too soon, or as a result of medicines given to the newborn.”*

(Czech Republic, information sheet)

*“Doing the second blood test does not mean that your baby is sick. The most common reason for repeated testing is that the first sample did not provide a clear conclusion. For example, because the baby was born prematurely or has not eaten enough yet.”*

(Hungary, leaflet)

5. Medical implications of screening

Most information products list the diseases that are screened for in the program and later describe them in more detail, such as the associated symptoms. The majority of the countries provide information about the diseases themselves, however, the amount of detail about certain diseases varies greatly between countries. Some countries do not provide information about the diseases, but do add a web link where information can be found. The United Kingdom was the only country where parents were offered a choice of which diseases can be screened for or not.

*“You can choose to have screening for SCD, CF or CHT individually, but can only choose to have screening for all six inherited metabolic diseases or none at all.” )*

(United Kingdom, booklet

6. Social implications of screening

Impact on daily life after being diagnosed with a rare disease is not addressed in the information products. The hereditary nature of many conditions in NBS is mentioned in twelve information products, without further explanation. A few information products explain about carrier status for the parents or siblings. The Netherlands and the United Kingdom mainly discuss the possibility of the disease presenting in a child when both parents are carriers. Some countries address the option for parents to choose whether they want to be informed about the carrier status. In France, the Netherlands, and the United Kingdom, sickle cell anemia carrier status can be reported to parents, sometimes as a secondary finding.

*“The heel prick can also show that your child is a carrier of sickle cell anaemia, but does not have the disease itself.”*

(Netherlands, booklet)

7. Financial implications of screening

Ten information products describe that the screening test is free of charge, as costs are often covered by health insurance or the government. Most countries that mention financial implications do this with a short, but comprehensive, statement.

*“The costs of the examination are covered by the statutory health insurance companies.”*

(Germany, booklet)

Although the United Kingdom government offers a free NBS program to parents, the information product also indicates that private companies provide screening tests outside of the program that are not free of charge. This can concern NBS, but also prenatal or other screening tests.

*“Some private companies also provide screening tests that you have to pay for.”*

(United Kingdom, booklet)

8. Follow-up plans including the availability of counselling and support services

Twenty countries summarize next steps for parents whose child has had a positive result in NBS. Possible treatments are often mentioned, however, the diagnostic pathway and clinical follow-up are often not explained. After a positive result, it is only indicated that more tests are needed to actually diagnose a disease. Czech Republic, Germany, and the United Kingdom are countries that clarify the fact that screening is not a definite diagnosis.

A child is often referred to a specialist for diagnostic testing. The support after diagnosis is described in half of the information products. A few times it is mentioned that the child is referred to a specialist. Even less often, reference is made to the specific specialist, such as an endocrinologist, geneticist, or a pediatrician.

*“Only if the diagnosis can be confirmed by intensive analyses, usually taking no more than a few days, treatment can begin immediately. Further steps concerning lifelong treatment are then planned together with the responsible paediatrician or the nearest paediatric clinic and specialists at the relevant centre for metabolic and hormone disorders.”*

(Switzerland, leaflet)

### 3.3. Other Relevant Themes in Parental Information Products

In addition to the eight knowledge aspects formulated by the GMC, the open coding showed additional items that we considered as relevant knowledge items.

- Storage of NBS material

Although it is very likely that many countries store NBS material, the information about the storage of the material is covered extensively by Denmark, Ireland, the Netherlands, Norway, Sweden, Switzerland, and the UK. A distinction in reasons for storage can be made between general information, quality control and test development, research, length, anonymity, and consent of parents. Some countries also include the reason why NBS material is stored.

*“Following screening a bloodspot may be used: 1) To check the results of the screen or to perform other tests recommended by your child’s doctor for which you as the parent must give consent. 2) For quality control purposes and to help improve the screening programme as approved by the HSE. In such circumstances all samples will be completely anonymized and it will not be possible to trace any results back to an individual child.”*

(Ireland, leaflet)

- Consent parents

In seventeen of the information products, it is stated that consent of parents is needed to participate in NBS. Both opt-in and opt-out systems are used for consent. In Croatia, Poland, and Hungary, participation in NBS is presented as mandatory in the information products.

*“The test is mandatory for all newborns in Hungary.”*

(Hungary, leaflet)

Another form of consent that has been addressed in some information products is consent of parents regarding the storage of the blood samples. This information often clarifies that the NBS material is stored anonymously in databases where only certain people can access the data for research purposes only. Frequently, an opt-out system is used, where parents need to sign if they object to the storage of NBS material.

*“We also request your consent to allow the blood sample and personal data to be used for quality assurance and to improve the screening programme. Participation in this is voluntary. Parents who do not wish to give their consent are not required to provide reasons for their decision, and there are no consequences for any treatment received by the baby.”*

(Norway, leaflet)

- Privacy and confidentiality

Eleven countries explain the importance of privacy and confidentiality regarding the NBS process. This includes information that is given about the privacy surrounding the results of NBS, storage of NBS material and the analysis of stored data.

*“Your child’s data will of course be kept strictly confidential.”*

(Austria, leaflet)

*“The results of the examinations is subject to medical confidentiality and may not be passed on to third parties without your consent.”*

(Germany, booklet)

- Performing the heel prick

Twenty-four information products briefly and succinctly discuss the way in which the screening test is performed. In addition, twenty-five products include the interval between birth and NBS.

*“The blood sample consists of a few drops of blood collected on blotting paper, taken by ‘pricking’ the skin on the outer side of one of the child’s heels.”*

(Denmark, leaflet)

*“Blood sampling is done between 48 and 72 hours after birth.”*

(Hungary, leaflet)

- Stakeholders

Twenty-one information products mention which stakeholders are in some way involved in the NBS process. Stakeholders that are mentioned are policy-makers on national and regional levels, the person who takes the blood sample, the person who does the analysis, parents, and possibly specialists from the hospital.

*“Early detection and early treatment for affected newborns are only possible if all those involved—parents, clinic or pediatrician and screening laboratory—work together without wasting time so that the test results are collected and checked in good time.”*

(Germany, booklet)

## 4. Discussion

### 4.1. Knowledge Aspects Are Well Presented

Parents are informed differently about NBS in Europe, and we aimed to study if these differences also mean a difference in knowledge aspects that are included in information products. This study found that in most countries, most knowledge aspects, as advised by the GMC, are included in the information products (Table 3). The purpose of screening, the likelihood of positive and negative findings, the uncertainties and risks attached to the screening process, medical implications of screening, and follow-up plans are described in most information products (knowledge aspects 1, 2, 4, 5, and 8). The possibility of false positive and false negative findings and the social and financial implications of NBS need more attention (knowledge aspects 3, 6, and 7). We would advise to include all eight knowledge aspects of the GMC in parental information, with additional NBS-specific knowledge aspects.

The results of this study also show that additional knowledge items may be relevant. These include describing the choice parents have to participate, how residual material is handled, how the heel prick is performed, what stakeholders are involved, and how privacy and confidentiality are safeguarded. The additional items that we found contribute further to the transparency of NBS programs. It has been shown that if parents are provided with adequate information about NBS, this enhances their trust and support for the program [25,26,27]. A risk of not providing adequate information lays in the larger impact of false positive results, but also in refusing to participate in screening which can lead to missed diagnoses [8].

While our study showed that most aspects of knowledge are presented as well as additional knowledge items, parents may not (fully) take in the information provided. Several studies have shown that parents often do not make an informed decision due to a lack of knowledge [11,15,19]. A possible explanation for this difference might be that parents make an undisputed choice; they see NBS as a routine procedure or do not often use the information provided to decide on participation. Healthcare providers such as screeners could verify whether parents understand the information and provide additional explanation if needed [19]. 

Van der Pal et al. (2010) discuss that the way of providing information or who provides the information may also influence the amount of knowledge of parents [19]. Moreover, multimedia tools and electronic platforms could have positive effects on the awareness and knowledge of parents when informed about NBS and use of residual dried blood spots [9]. However, more research is necessary to confirm the effectiveness of digital tools. 

### 4.2. Strengths and Limitations

To our knowledge, no previous research has been done into aspects of knowledge that are included in parental information products on NBS. A key strength of this study is that the GMC’s knowledge aspects are concise and used in other peer-reviewed studies [17,19,28,29]. Additionally, the open coding was conducted by multiple researchers, which reduces the chance of missing codes and the interpretation bias. We also achieved a 66% response rate, which represents a range of information products throughout Europe. 

However, some limitations of this study need to be addressed. This study has only assessed European information products, which means the sample may not reflect sufficient heterogeneity in how information is provided in different cultures. Moreover, this research is based on the available information products in 2018, and some information products have been updated since our data collection. Additionally, in some countries there is a tiered information provision, where extra information is provided by website links or distributed by healthcare professionals to parents who received a positive result. These additional means of communication were not included in our study and potentially include information, for example, on the social implications of a rare disease diagnosis. Furthermore, we did not assess the readability of the information products and when the information is provided. As these aspects are important for how parents receive information, we have included recommendations on future research.

### 4.3. Practical Recommendations and Future Research

The differences seen in the information products can inspire change to the information products countries have in place. There will be logical explanations why the information products are different on certain aspects, and a one-size fits all approach should not be strived for. Looking at the current information products, which contain a lot of information about diseases and possible treatments, a suggestion may be to focus less on detailed information of each disease and more on the other (additional) knowledge aspects in their products. To support equity in the information provided to help parents to make an informed decision about participation, we recommend using the knowledge aspects summarized here as a basis to evaluate or develop information products. 

Practical considerations will need to be made on how to convey the information. Initiatives are taken to involve parents when creating information material, to make it more in line with the wishes of parents [30,31,32]. Studies have already been conducted into the preferences of parents about how, when, where, and by whom the information should be provided [12,15,23,30,33]. For example, do parents have more interest in obtaining this through “paper” information products, video, health professionals, smartphone applications, or through social media? The “paper” information products could still be published, but for example contain more website links or QR codes to social media for additional information on certain topics. This keeps the information products as concise as possible, but if parents are interested, additional information is easy to find. In addition, information is always up-to-date this way, without having to adjust all products immediately after one small change of the program.

Moorhead et al. (2013) show that social media in health communication has many benefits, such as an increase in available, shared, and tailored information [34]. This may also provide opportunities to tailor information for parents from ethnic (sub)groups and with lower health literacy. It was further reported that parents prefer to receive NBS information during the pregnancy. A concrete suggestion was made by Fitzgerald et al. to provide the information during the waiting time before an appointment with the midwife. Information about NBS could be provided in the waiting rooms via multimedia channels; however, more research for this possibility is needed [23]. It is advisable to not overload parents with information, and make use of images and pictograms to illustrate the information provided [35]. Future research could focus on the minimal content that an information product should contain in relation to the ability to provide additional information via other means of communication. Specifically limitations of social media should be overcome before it can be safely used in daily screening routine [34]. 

Lastly, in the current study, European information products were analyzed, and a next step for further research could be to assess international information products for a broader interpretation of the knowledge aspects. Each country has its own standards, cultures, and values, and specific stakeholders involved in the policy-making process. By gaining insights into the international information provision, the information presented for different knowledge aspects will provide a broader range, and could anticipate better what a country needs in terms of knowledge about NBS. Countries that may have limited access to information of NBS can benefit. For example, research has been conducted into the possibility of starting NBS programs in India and African countries [4,36]. They might profit from an approachable format build on experience of countries with established NBS programs. To disclose the information collected in this study, we will make it available through the ISNS in a format with examples.

## 5. Conclusions

The findings of this study show that European information products cover many knowledge aspects regarding NBS. Some knowledge aspects could be improved to meet parents’ needs regarding NBS participation and follow-up plans, and additional relevant knowledge aspects were identified. The presentation of the information between countries differs, and how a good balance can be achieved between providing complete information while not overloading parents could be studied further. Including parents in the development of information products should be strived for. This should also focus on preferences of parents on alternative communication methods, which need to be considered and evaluated on their effectiveness. While our overview is a starting point for countries that want to change their information product, more research is needed to explore the needs of parents in all countries with different standards and cultures.

## Figures and Tables

**Table 1:** Knowledge aspects for making an informed decision about participation in screening programs proposed by the General Medical Council (GMC) [17], and our interpretation for the analysis (in italic).

Item	Knowledge
1	The purpose of screening *Describing the aim and importance of participation in NBS.*
2	The likelihood of positive and negative findings *Describing the possible results of NBS and/or explaining the probability of positive and negative findings.*
3	The possibility of false positive and false negative findings *Describing the chance of results that may be false positive or false negative for the conditions in the program.*
4	The uncertainties and risks attached to the screening process *Describing the uncertainties and risks of the screening test to indicate the reliability, such as an inconclusive result of NBS and no certainty that the baby is healthy.*
5	Medical implications of screening *Describing conditions in the program, giving information about the conditions, and describing possible treatment options.*
6	Social implications of screening *Describing how to deal with possible included (hereditary) diseases and emphasizing that there are certain risk groups for some diseases.*
7	Financial implications of screening *Describing possible costs for parents to participate in the NBS program based on the initial screening test, not on follow-up testing and/or treatment.*
8	Follow-up plans including the availability of counselling and support services *Describing next steps after parents have received a positive result in NBS and the support after diagnosis.*

GMC: General Medical Council; NBS: newborn screening.

**Table 2:** Characteristics information products in different European countries. ^1^

Country	Information Product	Language	Number of Pages
Austria	Leaflet	Native	2
Croatia	Booklet	Native	5
Cyprus	Information sheet	English	2
Czech Republic	Information sheet	English	2
Denmark	Leaflet	English	4
Estonia	Booklet	Native	5
France	Booklet	Native	10
Germany	Booklet	Native	6
Hungary	Leaflet	Native	2
Iceland	Leaflet	Native	2
Ireland	Leaflet	English	3
Italy ^2^	Leaflet	Native	4
Lithuania	Information sheet	English	2
Luxembourg	Booklet	English	8
Malta	Information sheet	English	1
Netherlands	Booklet	English	10
North Macedonia	Leaflet	Native	4
Norway	Leaflet	English	2
Poland	Leaflet	Native	2
Portugal	Leaflet	English	6
Romania	Information sheet	Native	1
Serbia	Leaflet	Native	2
Spain ^3^	Leaflet	Native	2
Sweden	Information sheet	English	1
Switzerland	Leaflet	English	4
United Kingdom	Booklet	English	12

^1^ The information products presented in this table represent the products we received in 2018, a number of countries have updated their information since then, so these indicators may not always represent the current situation for all countries. ^2^ Information product from the region Tuscany, Italy. No national information product was obtained. ^3^ Information product from the region Andalusia, Spain. No national information product was obtained.

**Table 3:** Key results of all knowledge aspects per country ^1^.

Aspect of Knowledge	1. Purpose of NBS	2. Positive or Negative Findings	3. False Positive or False Negative Findings	4. Uncer-Tainties and Risks	5. Medical Implications	6. Social Implications	7. Financial Implications	8. Follow-Up Plans	Total
Austria	✓		✓	✓	✓	✓	✓	✓	7
Croatia	✓	✓	✓	✓	✓	✓		✓	7
Cyprus	✓				✓	✓		✓	4
Czech Republic	✓	✓	✓	✓	✓			✓	6
Denmark	✓			✓	✓			✓	4
Estonia	✓	✓	✓	✓	✓		✓	✓	7
France	✓	✓		✓	✓	✓			5
Germany	✓	✓		✓	✓	✓	✓	✓	7
Hungary	✓			✓	✓	✓		✓	5
Iceland	✓	✓	✓	✓				✓	5
Ireland	✓	✓	✓	✓		✓		✓	6
Italy	✓	✓		✓	✓		✓	✓	6
Lithuania	✓	✓		✓	✓		✓	✓	6
Luxembourg	✓	✓			✓				3
Malta	✓	✓		✓				✓	4
Netherlands	✓	✓	✓	✓	✓	✓	✓	✓	8
North Macedonia	✓				✓				2
Norway	✓	✓	✓	✓	✓			✓	6
Poland	✓	✓		✓	✓	✓	✓	✓	7
Portugal	✓			✓	✓				3
Romania	✓					✓	✓		3
Serbia	✓	✓						✓	3
Spain	✓	✓		✓				✓	4
Sweden	✓	✓		✓	✓				4
Switzerland	✓	✓			✓	✓	✓	✓	6
United Kingdom	✓	✓	✓	✓	✓	✓	✓	✓	8
Total	26	19	9	20	20	12	10	20	

^1^ The information products presented in this table represent the products we received in 2018, a number of countries have updated their information since then, so these indicators may not always represent the current situation for all countries.
